# Long-term data set of small mammals from owl pellets in the Atlantic-Mediterranean transition area

**DOI:** 10.1038/sdata.2016.85

**Published:** 2016-09-27

**Authors:** Nora Escribano, David Galicia, Arturo H. Ariño, Carmen Escala

**Affiliations:** 1 Department of Environmental Biology, University of Navarra, Irunlarrea 1, Pamplona 31008, Spain

**Keywords:** Zoology, Biodiversity

## Abstract

We describe the pellet sampling data set from the Vertebrate Collection of the Museum of Zoology of the University of Navarra. This data set compiles all information about small mammals obtained from the analysis of owl pellets. The collection consists on skulls, mandibles, and some skeletons of 36 species of more than 72,000 georeferenced specimens. These specimens come from the Iberian Peninsula although most samples were collected in Navarra, a highly diverse transitional area of 10,000 kilometre square sitting across three biogeographical regions. The collection spans more than forty years and is still growing as a result of the establishment of a barn owl pellet monitoring network in 2015. The program will provide critical information about the evolution of the small mammals’ community in this transition zone as it changes over time.

## Background & Summary

Natural history collections are in the spotlight of scientific community as they provide long-term data of species distribution all around the world^1^. These data sets that cover long periods of time are valuable as they are key in understanding and documenting biodiversity, providing a base point in time that allow comparison between previous conditions and current ones^2–4^. Many areas of scientific research such as climate change ecology, biodiversity distribution and invasive species rely on these biological records found in natural history collections^5–7^. In fact, collections are usually composed of voucher specimens which are valuable now and in coming years as they can be revised, verified and re-used in the future^2,8^. However, large portions of the vast amount of collections that could be digitalized and shared via repositories like GBIF, DRYAD or FIGSHARE are still locked away^9–11^ and the access to this enormous information is still complicated^12^. Therefore, it is essential that we bring these natural history collections to light making them accessible for the wider research community, allowing the assessment of current and prediction of future status of the biodiversity apparently being compromised^13,14^.

The Museum of Zoology of University of Navarra (MZNA) was established in 1980 to safeguard material from the research carried out in the former department of Zoology and Ecology and now Environmental Biology department^15^. It is a Data Provider for the Global Biodiversity Information Facility (GBIF) and curates collections of vertebrates (see e.g., refs 16,17) and invertebrates^18^. One of its most complete data sets in terms of taxonomic, temporal and spatial coverage is the ‘Pellet sampling’ data set (Data Citation 1) included in the Vertebrate Collection of MZNA^19^. This data set includes information about small mammals' records obtained from the analysis of barn owl pellets. It refers skulls, mandibles and skeletons of more than 72,000 georeferenced specimens belonging to 36 species. From 1967 on, this collection increased in terms of number of species, specimens and spatial coverage, and continues to grow as a result of the establishment in 2015 of a monitoring network based on pellet sampling. The monitoring program is designed to provide new data at least twice a year increasing temporal resolution and allowing the assessment of seasonal trends in the small mammals’ community.

Specimens in this data set come from a heterogeneous area where Alpine, Atlantic and Mediterranean biogeographical regions^20^ come in contact. The North of Navarra is influenced by the Atlantic Ocean and the Pyrenean Range, whereas as we are move South the region turns into Mediterranean conditions within less than 100 km, with a complete turnover in the flora and fauna communities. Highly diverse climate coupled to complex topography make this region a highly interesting area for studying the transition between Eurosiberian species (from Atlantic and Alpine biogeographical regions) to Mediterranean ones.

The coincidence of a small but highly diverse biogeographical area and a long-term data set well represented across that very area allows for meaningful studies on the distribution patterns of the small mammals’ community. It also provides an opportunity to assess possible changes in species distributional ranges as a result of the ongoing environmental changes^13^. Navarra is subject to significant changes in land use^21^ that can affect the composition of its small mammals’ community, as reported in other areas both in Europe and worlwide^22,23^. Long-term series of data like this data set can bring to light shifts in the distributional ranges of species coping with warmer conditions, land uses changes or invasive species^4,22,24^.

## Methods

### Geographic location

The ‘Pellet sampling’ data set contains specimens from the Iberian Peninsula but most of the records come from the province of Navarra (>70%). Thus, hereinafter we will refer to Navarra as the study extent of the database.

**Figure 1:**
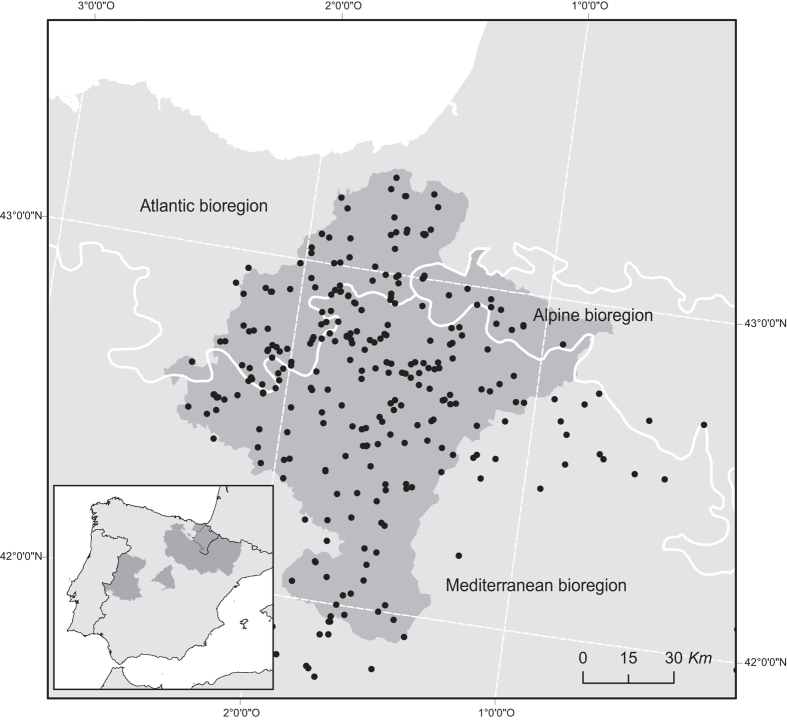
Spatial coverage of ‘pellet sampling’ dataset. Black dots: Sampling localities within Navarra (outlined in grey) and other provinces holding small mammals’ records (grey areas, inset). Biogeographical regions are delimited by white lines.

Navarra is located in the north of Spain and covers an area of 10,391 km^2^ (Fig. 1). It is located between the western end of the Pyrenees and the Ebro Basin, including Alpine, Atlantic and Mediterranean biogeographical regions inside its territory. Due to its location and topography, Navarra is characterized by a wide range of climate conditions varying from Oceanic to Mediterranean, and high landscape diversity is associated with these particular conditions^25,26^. The northern half of Navarra reaches mean altitudes above 800 m (range 200–2,000 m), 1,200–1,700 mm mean annual precipitation and moderate temperatures. Its landscape is dominated by pastures and beech and oak forests as well as pine repopulation for wood exploitation. Further South, climate and landscape shift to altitudes below 400 m and lower rainfall (<400 mm). The high diversity of flora and fauna in Navarra derives from this transition from Eurosiberian to Mediterranean region in a relatively small distance of 160 km along the north-south axis^26^, and as a result of the variety of habitats the small mammals’ community in Navarra, which includes 31 of the 34 species of native insectivorous and rodents cited in the Iberian Peninsula^27^, is also highly diverse.

### Field surveys

Specimens were obtained from barn owl pellets. This method has been proven to give a useful picture of the small mammals’ community^28,29^. Pellets were collected in places commonly used by barn owls such as nests and roosts found in churches and barns^30^. When a locality was positive for pellets, all pellets were collected.

More systematic surveys were fielded during the acquisition of data for a regional mammal atlas. In this study, Navarra was gridded with 10×10 km cells. Sampling sites were searched for potential localities holding barn owls nests or roosts. These places were usually bell towers and barns. However, there have been additional contributions from different sources such as research projects, PhD researches, donations, occasional sampling, volunteers and student’s works.

### Laboratory work

All pellets brought to the laboratory were frozen at −20 °C for at least a week to remove bugs and then dried at 50 °C to avoid fungal growth. Afterwards, pellets were stored in plastic bags indicating sampling site and the date until they were processed.

Once dried, pellets were dissected separating bones from the remaining fur and feathers. Skulls and mandibles were preserved while other remains were discarded. Specimens were identified to species level when possible using suitable literature^31^. If key characters were missing (e.g., teeth), specimens were identified to genus level. Mandibles found separately from skull were stored as well and identified to family level. After identification, data was digitalized in the MZNA database and the specimens were sealed in zip plastic bags labelled with their accession number and stored in the MZNA collection facility. Specimens consisting on skull and mandibles were stored together.

### Data management and standardization

All specimens were systematically recorded in the MZNA database system (Zootron v.4)^32^.

This collection is accessible through IPT portal from GBIF. We carried out a major effort for standardize the records to within the DarwinCore Terms^33^. We used 39 controlled terms to describe the information contained in the data set: id, modified, institutionCode, collectionCode, basisOfRecord, occurrenceID, catalogNumber, otherCatalogNumbers, recordedBy, individualCount, sex, preparations, disposition, eventDate, verbatimEventDate, continent, island, country, stateProvince, locality, verbatimElevation, minimumElevationInMeters, maximumElevationInMeters, verbatimCoordinates, decimalLatitude, decimalLongitude, geodeticDatum, CoordinateUncertaintyInMeters, identifiedBy, scientificName, kingdom, phylum, class, order, family, genus, specificEpithet, taxonRank, scientificNameAuthorship. For all definitions of these terms see^33^.

The term *eventDate* must be interpreted as the date when pellets were collected, not as the date of the capture of the prey which is unknown although it lies between consecutive pellet collection events for a locality. When a nest or roost is sampled, the pellets found are the result of accumulation of hunting events by the raptor. The batch number can be found under the term *otherCatalogNumbers*.

## Data Records

The *pellet sampling* data set is hosted by the Global Biodiversity Information Facility and can be found in one.txt file (Data Citation 1) in DarwinCore standards of GBIF^33^. The data descriptor we present here corresponds to version 2.4 of this data set.

**Figure 2:**
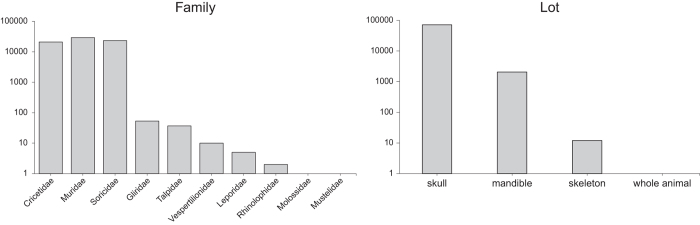
Distribution of 73,574 records by families and type of lot (log scale).

The data set is an occurrence-based table that contains 73,574 records of small mammals derived from the analysis of barn owl pellets (see also Fig. 2). More than the 99% of the records are georeferenced (73,474 records) and 73,316 records (99.6%) include at least the collection date with year resolution. However, the number of ‘complete’ records -those that reach species level, include full coordinates and complete day date (year, month and day) are 48,003 (65.2%).

The data set includes 5 orders, 10 families and 36 species. Rodents and soricomorphs make up most of the collection. Muridae and Soricidae are the most abundant families in the data set (Fig. 2), being 39.66 and 31.67% respectively, followed by Cricetidae with 28.51% of the total records. Information about bats, lagomorphs and carnivores is scarce, representing less than 1% of the collection (for more details in taxonomic coverage see Tables 1 and 2). Skulls and mandibles dominate the collection deposited in MZNA.

The resources of this data set are diverse including systematic surveys for the regional atlas, monitoring network, thesis projects, students work, occasional pellet sampling, donations and volunteer contributions. This variety of resources translated into varying completeness of the data, as each resource was fit for its particular purpose; but the aggregation of the records into the data set was designed to maintain their fitness-for-use (FFU) throughout^34^.

**Figure 3:**
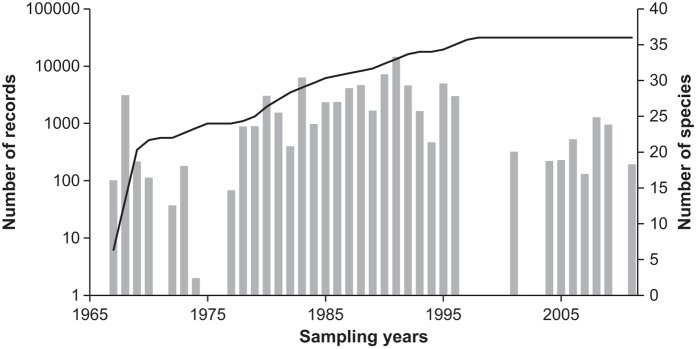
Historical inventorying of 73,316 records of small mammals in the ‘pellet sampling’ data set that include collection year. Grey bars represent number of records gathered each year (log scale). The black line is the species accumulation curve.

Most of the specimens come from the north of Iberian Peninsula (Fig. 1) and date from 1967 to the present time. The way in which data has accumulated over time is shown in Fig. 3. The years prior to the elaboration of a regional small mammals’ atlas^27^ peak between 1987 and 1992. Another period of constant sampling effort was the early 80’s. There is a gap after 1996. However, the establishment of the monitoring network in 2015 is being providing new sampling sites and specimens to the collection every year, increasing the temporal coverage and the possibility of use of this data in global change ecology, and changing distributional patterns or species distributional models. Data derived from this monitoring network will be updated annually.

## Technical Validation

While standardizing the records to DarwinCore standards, we carried out a major effort for checking errors and inconsistencies among the data. We followed the guidelines proposed by Chapman^35^ and checked taxonomical, geographical and temporal errors in the data set.

Most specimen identifications were verified by Dr Escala, professor of Zoology, the main responsible for the creation of the collection. Other experienced researchers have also contributed to the identification of specimens from this collection.

Scientific names were updated when necessary to keep up with current taxonomy according to Mammal Species of the World and the Atlas of Mammals from the Iberian Peninsula^36,37^.

Various coordinate formats were found during the standardization of the data. Most localities were originally georeferenced within the UTM projection system and noted down as military grid coordinates, although more recently digitalized records already used geographic coordinates. However, as a requirement of DarwinCore standards, all coordinates in UTM/MGRS were transformed to geographic coordinates. Coordinate uncertainty was calculated using the point-radius method and given in meters. Verbatim coordinates are preserved in the data set and can be used as well. Finally, we checked locality consistency by visual inspection overlapping then against an administrative map using GIS^38^. We also searched coordinates in a gazetteer for localities that lacked this geographical information.

Temporal errors were checked. Collecting dates format was transformed to the ISO 8601 format (i.e., YYYY-MM-DD).

## Additional Information

**How to cite this article:** Escribano, N. *et al.* Long-term data set of small mammals from owl pellets in the Atlantic-Mediterranean transition area. *Sci. Data* 3:160085 doi: 10.1038/sdata.2016.85 (2016).

## Supplementary Material



## Figures and Tables

**Table 1:** Taxonomic coverage of pellet sampling dataset and number of records of each species.

**Order**	**Family**	**Species**	**Records**
Rodentia	Cricetidae	*Arvicola sapidu*s Miller, 1908	207
		*Arvicola terrestris* (Linnaeus, 1758)	179
		*Chionomys nivalis* (Martins, 1842)	1
		*Microtus agrestis* (Linnaeus, 1761)	3606
		*Microtus arvalis* (Pallas, 1779)	525
		*Microtus cabrerae* Thomas, 1906	60
		*Microtus duodecimcostatus* (De selys-longchamps, 1839)	10270
		*Microtus gerbei* (Gerbe, 1879)	1409
		*Microtus lusitanicus* (Gerbe, 1879)	2872
		*Myodes glareolus* (Schreber, 1780)	350
	Gliridae	*Eliomys quercinus* (Linnaeus, 1766)	41
		*Glis glis* (Linnaeus, 1766)	11
	Muridae	*Apodemus flavicollis* (Melchior, 1834)	553
		*Apodemus sylvaticus* (Linnaeus, 1758)	11747
		*Micromys minutus* (Pallas, 1771)	70
		*Mus domesticus* (Rutty, 1772)	830
		*Mus spretus* Lataste, 1883	13987
		*Rattus norvegicus* (Berkenhout, 1769)	211
		*Rattus rattus* (Linnaeus, 1788)	35
Soricomorpha	Soricidae	*Crocidura russula* (Hermann, 1780)	17124
		*Crocidura suaveolens* (Pallas, 1811)	34
		*Neomys anomalus* Cabrera, 1907	42
		*Neomys fodiens* Pennant, 1771	311
		*Sorex araneus* Linnaeus, 1758	2
		*Sorex coronatus* Millet, 1828	3775
		*Sorex granarius* Miller, 1910	1
		*Sorex minutus* Linnaeus, 1776	602
	1	*Suncus etruscus* (Savi, 1822)	1166
	Talpidae	*Talpa europaea* Linnaeus, 1758	22
		*Talpa occidentalis* Cabrera, 1907	1
Chiroptera	Rhinolophidae	*Rhinolophus ferrumequinum* Schreber, 1774	1
		*Rhinolophus hipposideros* Bechstein, 1800	1
	Vespertilionidae	*Myotis myotis* Borkhausen, 1797	3
		*Pipistrellus kuhlii* Natterer, 1819	1
		*Pipistrellus pipistrellus* Schreber, 1775	3
		*Plecotus austriacus* Fischer, 1829	2
	Molossidae	*Tadarida* sp	1
Lagomorpha	Leporidae	*Lepus* sp	2
		*Oryctolagus cuniculus* (Linnaeus, 1758)	3
Carnivora	Mustelidae	*Mustela nivalis* Linnaeus, 1758	1

**Table 2:** Taxonomic coverage of the pellet sampling data set (73,574 records) and number of records per taxa.

**Order**	**Family**	**Taxa**	**Records**
Rodentia	Cricetidae	*Arvicola* sp	12
		*Arvicola sapidus* Miller, 1908	207
		*Arvicola terrestris* (Linnaeus, 1758)	179
		*Chionomys nivalis* (Martins, 1842)	1
		Cricetidae	619
		*Microtus* sp	869
		*Microtus agrestis* (Linnaeus, 1761)	3606
		*Microtus arvalis* (Pallas, 1779)	525
		*Microtus cabrerae* Thomas, 1906	60
		*Microtus duodecimcostatus* (De selys-longchamps, 1839)	10270
		*Microtus gerbei* (Gerbe, 1879)	1409
		*Microtus lusitanicus* (Gerbe, 1879)	2872
		*Myodes glareolus* (Schreber, 1780)	350
	Gliridae	*Eliomys quercinus* (Linnaeus, 1766)	41
		Gliridae	1
		*Glis glis* (Linnaeus, 1766)	11
	Muridae	*Apodemus* sp	215
		*Apodemus flavicollis* (Melchior, 1834)	553
		*Apodemus sylvaticus* (Linnaeus, 1758)	11747
		*Micromys minutus* (Pallas, 1771)	70
		Muridae	784
		*Mus* sp	721
		*Mus domesticus* (Rutty, 1772)	830
		*Mus spretus* Lataste, 1883	13987
		*Rattus* sp	27
		*Rattus norvegicus* (Berkenhout, 1769)	211
		*Rattus rattus* (Linnaeus, 1788)	35
Soricomorpha	Soricidae	*Crocidura* sp	99
		*Crocidura russula* (Hermann, 1780)	17124
		*Crocidura suaveolens* (Pallas, 1811)	34
		*Neomys* sp	16
		*Neomys anomalus* Cabrera, 1907	42
		*Neomys fodiens* Pennant, 1771	311
		*Sorex* sp	19
		*Sorex araneus* Linnaeus, 1758	2
		*Sorex coronatus* Millet, 1828	3775
		*Sorex granarius* Miller, 1910	1
		*Sorex minutus* Linnaeus, 1776	602
		Soricidae	115
		*Suncus etruscus* (Savi, 1822)	1166
	Talpidae	*Talpa* sp	14
		*Talpa europaea* Linnaeus, 1758	22
		*Talpa occidentalis* Cabrera, 1907	1
Carnivora	Mustelidae	*Mustela nivalis* Linnaeus, 1758	1
Chiroptera	Molossidae	*Tadarida* sp	1
	Rhinolophidae	*Rhinolophus ferrumequinum* Schreber, 1774	1
		*Rhinolophus hipposideros* Bechstein, 1800	1
	Vespertilionidae	*Myotis myotis* Borkhausen, 1797	3
		*Pipistrellus* sp	1
		*Pipistrellus kuhlii* Natterer, 1819	1
		*Pipistrellus pipistrellus* Schreber, 1775	3
		*Plecotus austriacus* Fischer, 1829	2
Lagomorpha	Leporidae	*Lepus* sp	2
		*Oryctolagus cuniculus* (Linnaeus, 1758)	3
